# Expression of a Truncated Yeast Ccc1 Vacuolar Transporter Increases the Accumulation of Endogenous Iron

**DOI:** 10.3390/genes12081120

**Published:** 2021-07-23

**Authors:** Raquel Sorribes-Dauden, María Teresa Martínez-Pastor, Sergi Puig

**Affiliations:** 1 Departamento de Bioquímica y Biología Molecular, Universitat de València, Doctor Moliner 50, 46100 Burjassot, Valencia, Spain; Raquel.Sorribes@uv.es; 2 Departamento de Biotecnología, Instituto de Agroquímica y Tecnología de Alimentos (IATA), Consejo Superior de Investigaciones Científicas (CSIC), Agustín Escardino 7, 46980 Paterna, Valencia, Spain

**Keywords:** yeast, *Saccharomyces cerevisiae*, iron, Ccc1

## Abstract

Iron is an essential micronutrient for all eukaryotic organisms because it participates as a redox cofactor in multiple metabolic processes. Iron bioavailability is highly restricted due to the low solubility of its oxidized form, frequently leading to iron deficiency anemia. The baker’s yeast *Saccharomyces cerevisiae* is used as a model organism for iron homeostasis studies, but also as a food supplement and fermentative microorganism in the food industry. Yeast cells use the vacuolar Ccc1 transporter to detoxify and store excess iron in the vacuoles. Here, we modulate *CCC1* expression and properties to increase iron extraction from the environment. We show that constitutive expression of full-length *CCC1* is toxic, whereas deletion of its cytosolic amino-terminal (Nt) domain (*NtΔCCC1*) rescues this phenotype. Toxicity is exacerbated in cells lacking *AFT1* transcription factor. Further characterization of NtΔCcc1 protein suggests that it is a partially functional protein. Western blot analyses indicate that deletion of Ccc1 Nt domain does not significantly alter GFP-Ccc1 protein stability. A functional full-length GFP-Ccc1 protein localized to particular regions of the vacuolar membrane, whereas GFP-NtΔCcc1 protein was evenly distributed throughout this endogenous membrane. Interestingly, expression of *NtΔCCC1* increased the accumulation of endogenous iron in cells cultivated under iron-sufficient conditions, a strategy that could be used to extract iron from media that are not rich in iron.

## 1. Introduction

Iron is an essential micronutrient for all eukaryotic organisms. Its redox and coordination properties allow iron to participate as a cofactor in a wide range of metabolic processes including the synthesis of DNA, proteins, and lipids, mitochondrial respiration and photosynthesis, and oxygen transport. Despite being one of the most abundant elements in the Earth’s crust, iron bioavailability is extremely restricted in aerobic environments as it is mostly present in its oxidized from (Fe^3+^), which is highly insoluble at physiological pH. According to the World Health Organization, iron deficiency anemia (IDA) is one of the most prevalent human nutritional disorders (even present in developed countries) with high impact and potentially fatal health consequences in children, women of fertile age, pregnant women, and the elderly (reviewed in [1,2]). Consequences of IDA include diminished learning ability and poor physical growth of infants, fatigue and reduced work productivity in adults, greater susceptibility to infections, and increased risk of death associated with pregnancy and childbirth. Multiple strategies are used to prevent and treat IDA including diversification of the diet and supplementation or food fortification with particular iron forms (reviewed in [3]).

The baker’s yeast *Saccharomyces cerevisiae* contributed significantly to the identification and characterization of many components of the human, plant, and fungal iron homeostatic networks. Moreover, this yeast is used in multiple biotechnological applications including fermentative processes to obtain different food products (e.g., bread, wine, and beer), as a nutritional supplement since it is rich in proteins, vitamins, and fiber, and as a factory for the production of compounds of interest. Iron-enriched yeasts are currently being evaluated as promising sources of iron to prevent and palliate iron deficiency in humans and animals [4]. In fact, iron-enriched baker’s yeasts are efficient in helping animals to recover from iron deficiency [5]. However, when present in excess, iron can engage in Fenton redox reactions that promote the formation of reactive oxygen species (ROS), which damage cells at the level of proteins, lipids, and DNA [6]. Therefore, yeast cells must coordinate iron acquisition, distribution, utilization, detoxification, and storage.

**Figure 1:**
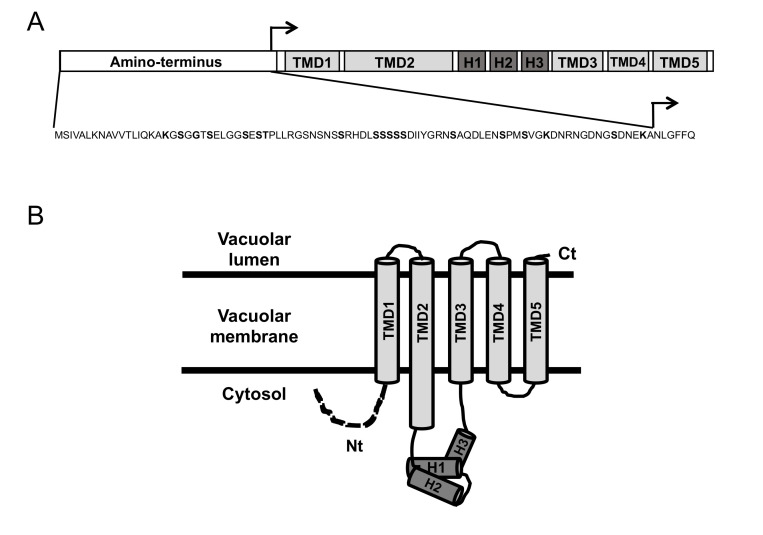
Schematic representation of Ccc1/VIT1 domain organization (**A**) and membrane topology (**B**), based on EgVIT1 structure and protein alignments [11,12]. A and B: Grey rectangles/cylinders represent transmembrane domains (TMDs); dark grey rectangles/cylinders represent three helices. A: White rectangles represent the Nt domain, whose amino acid sequence is indicated below. Bold letters indicate phosphorylated and ubiquitylated residues, as well as mutation G22R in Malaysian yeast strains [13,14,15]. Black arrow indicates where NtΔCcc1 protein starts.

Opposite to animals, yeast cells do not express ferritin; instead they accumulate iron in the vacuole for detoxification and storage purposes [7]. Iron can enter yeast vacuoles by endocytosis and via Ccc1, a protein that localizes to the vacuolar membrane and transports iron into its lumen [8,9]. Since yeast cells cannot excrete iron, Ccc1 constitutes the main yeast iron detoxification and resistance factor. Thus, yeast cells lacking *CCC1* (*ccc1Δ* mutants) are highly sensitive to elevated levels of environmental iron [8,10]. With the exception of animals, Ccc1 homologs are present in many organisms, including bacteria, protists, fungi, and plants, where they are denoted VIT1 proteins (reviewed in [11]). The resolution of the crystal structure of *Eucalyptus grandis* VIT1 ortholog (EgVIT1) made possible the identification of essential and conserved motifs required for metal transport, as well as other species-specific domains [11,12]. According to EgVIT1 structure and protein alignments, Ccc1/VIT1 proteins, including yeast Ccc1, possess five transmembrane domains (TMDs), a metal-binding loop containing three alpha helixes (H1–H3) between TMD2 and TMD3 that faces the cytosol, a cytosolic amino-terminal (Nt) region, and a vacuolar carboxyl terminus (Figure 1) [12]. Structure-function data in EgVIT1 also suggest that Ccc1/VIT1 proteins dimerize and function as H^+^ antiporters for Fe^2+^ [12]. The Ccc1/VIT1 cytosolic Nt domain is not conserved and displays different properties depending on the species [11]. Yeast possesses a cytosolic Ccc1 Nt with serine and lysine residues that have been identified in proteomic studies as phosphorylated and ubiquitylated, respectively [13,14]. Moreover, Malaysian wild *S. cerevisiae* strains harboring a mutation in their Nt region (G22R) are sensitive to high iron concentrations [15]. Thus, although according to conservation features the Nt domain of yeast Ccc1 seems dispensable, it may be important for regulatory functions.

Multiple transcription factors contribute to upregulation of *CCC1* mRNA levels in response to iron excess (reviewed in [11,16,17]). A member of the yeast activator protein (Yap) subfamily of transcriptional factors denoted Yap5 associates to specific *cis* elements within *CCC1* promoter and activates its transcription in response to iron excess [10,18,19,20]. Moreover, the low-glucose sensor Snf1 also contributes to *CCC1* mRNA upregulation in high iron conditions by activating the general stress transcription factors Mns2 and Msn4 [21]. In response to iron deficiency, yeast Aft1 and Aft2 transcription factors activate iron acquisition via the Fet3-Ftr1 high-affinity iron transport system, iron mobilization from the vacuole, and a metabolic remodeling mediated by the mRNA-binding protein Cth2 that post-transcriptionally limits *CCC1* expression [17,18,22,23,24].

Previous studies demonstrated that *CCC1* overexpression promotes iron uptake and accumulation into the vacuole [8,9,25]. Although this strategy seems appropriate to extract iron from the environment and enrich yeast cells with iron, we show here that constitutive expression of *CCC1* is toxic to yeast cells cultivated in iron replete conditions. Interestingly, deletion of the Ccc1 Nt domain rescues toxicity, allowing a more efficient extraction of iron from the surrounding milieu. A potential function of Ccc1 Nt is discussed.

## 2. Materials and Methods

### 2.1. Yeast Strains

Yeast strains used in this study (Invitrogen) include BY4741 (MATa, his3Δ1, leu2Δ0, met15Δ0, ura3Δ0), BY4743 (MATa/α his3Δ1/his3Δ1 leu2Δ0/leu2Δ0 LYS2/lys2Δ0 met15Δ0/MET15 ura3Δ0/ura3Δ0), ccc1Δ (BY4741 ccc1::KanMX6), ccc1Δ (BY4743 ccc1::KanMX6/ccc1::KanMX6), fet3Δ (BY4741 fet3::KanMX6), ftr1Δ (BY4741 ftr1::KanMX6), aft1Δ (BY4741 aft1::KanMX6), and cth2Δ (BY4741 cth2::KanMX6).

### 2.2. Plasmids

Plasmids used in this work include empty vectors p416TEF [26], p416GAL, and pUG36 (kindly provided by U. Güldener and J. H. Hegemann, Heinrich Heine University Düsseldorf, Germany), and the corresponding vectors with either *CCC1* or *NtΔCCC1* (lacking the first 87 Nt amino acids) coding sequences cloned into BamHI and XhoI. The pUG36 vector expresses either *CCC1* or *NtΔCCC1* coding sequences under the control of the methionine-regulated *MET17* promoter and adds a GFP tag at the Nt end.

### 2.3. Growth Conditions

Yeasts were cultivated at 30 °C in synthetic complete (SC) medium lacking uracil (SC-Ura) or lacking both uracil and methionine (SC-Ura-Met). Glucose was substituted with galactose in SC-Ura/Galactose medium. Iron was added to SC media at the indicated concentrations from a 100 mM Fe(NH_4_)_2_(SO_4_)_2_ (FAS, Merck-Sigma) stock solution. Stock solutions were prepared in 0.1M HCl to facilitate iron solubility and were used fresh. A control with the equivalent HCl concentration was always prepared. The Fe^2+^-specific chelator ferrozine (Fz, Merck-Sigma) was added to SC plates from a 100 mM stock solution freshly prepared in water. To grow yeast cells in liquid media, SC-Ura or SC-Ura-Met precultures were reinoculated at an optical density at 600 nm (OD_600nm_) of 0.2 and then incubated at 30 °C and 190 rpm in 50 mL flasks overnight. Alternatively, for duplication time measurements, yeast cells were inoculated in 96-well plates at an OD_600nm_ of 0.2 in 270 μL of liquid SC-Ura medium with 500 μM FAS, and the OD_600nm_ was determined in a Spectrostar Nano absorbance microplate reader (BMG Labtech, Ortenberg, Germany) every 30 min for 36 h at 28 °C. Duplication times were calculated using the online tool GCAT (Growth Curve Analysis Tool) as described in [27]. To perform growth assays in solid media, cells from overnight precultures were diluted to an OD_600nm_ of 0.1 and then spotted directly and after 1:10 and 1:100 dilutions in sterile water. Plates were incubated at 28 °C for 3 to 6 days and were then photographed.

### 2.4. Iron Measurements

Yeast cells were collected and treated as previously described, and a colorimetric assay was used to determine the endogenous levels of iron [28,29]. To determine yeast dry weight (DW), cells corresponding to a total OD_600nm_ x mL of 30 were collected, washed, transferred to a previously weighed Eppendorf tube, and dried at 50 °C for 3 days. Finally, yeast DW was obtained by subtracting the total weight from the Eppendorf tube weight. To determine the iron that yeast cells extracted from the surrounding medium, endogenous iron levels were divided by the volume of medium in which these cells were growing.

### 2.5. RNA Analyses

To determine the expression levels of particular mRNAs, total RNA was extracted and reverse transcription-quantitative PCR (RT-qPCR) was performed as previously described [30]. *ACT1* mRNA values were used to normalize data. Data and error bars represent the averages and standard deviations from three independent biological experiments. Specific primer pairs for *FTR1*, *CTH2*, and *ACT1* were used.

### 2.6. Protein Analyses

To determine Ccc1 and NtΔCcc1 protein stability, exponentially growing cells were cultivated in SC-Ura-Met without (iron-sufficient conditions) or with 3 mM FAS (high-iron conditions) for 1 h (modified from [31]). *CCC1* synthesis was halted by adding methionine to a final concentration of 3 mM and cells were harvested at the indicated time points. Yeast proteins were extracted by the alkali method and processed and analyzed by western blot, as previously detailed [32,33]. Anti-GFP (Roche) and anti-Pgk1 (Thermo Fisher Scientific, Waltham, MA, USA) were used to detect GFP-Ccc1/GFP-NtΔCcc1 and Pgk1 proteins, respectively. 

### 2.7. Fluorescence Microscopy

Images were captured on an Eclipse 90i Nikon microscope (Nikon Corporation, Tokio, Japan) using a digital camera (Nikon DS-5Mc, Tokio, Japan). To stain vacuolar membranes, cells were incubated for 15 min in 8 μM of FM4-64, washed, and incubated for an additional hour.

### 2.8. Statistical Analyses

Tailed T-student tests were used to determine statistical significance. Significant differences (*p*-value < 0.05) are indicated by different letters, whereas at least one letter being the same indicates that there are no significant differences.

## 3. Results

### 3.1. The Toxicity of a Constitutively Expressed CCC1 Is Rescued by Iron Addition

**Figure 2:**
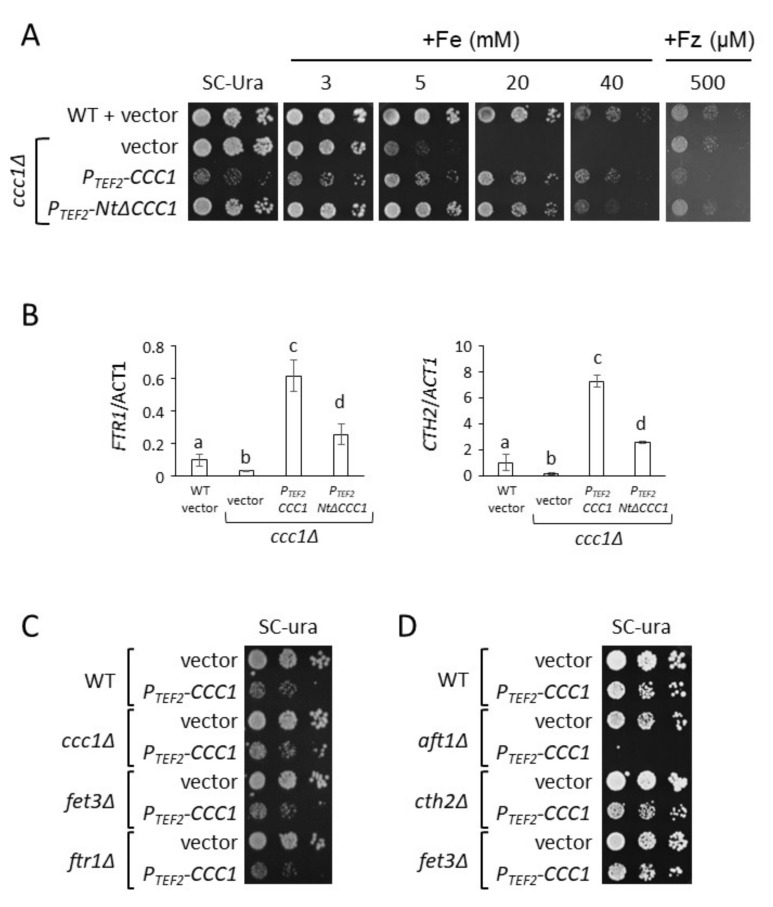
Toxicity of *CCC1* constitutive expression under iron-sufficient conditions is rescued by iron addition or by deleting the Ccc1 cytosolic amino-terminal region. BY4741 (WT) cells were transformed with p416TEF (vector), and *ccc1Δ* cells were transformed with p416TEF (vector), p416TEF-CCC1 (*P_TEF2_-CCC1*), and p416TEF-NtΔCCC1 (*P_TEF2_-NtΔCCC1*) plasmids. (**A**) Expression of *CCC1* altered growth in media with different iron availability. Cells were cultivated overnight in SC-Ura and then spotted on SC-Ura media containing different concentrations of FAS (Fe) or ferrozine (Fz). (**B**) *FTR1* and *CTH2* mRNA levels. Yeast cells were cultivated in SC-Ura + 500 μM FAS for 6 h. Total RNA was extracted and the levels of particular mRNAs were determined by RT-qPCR. *ACT1* was used to normalize data. Three independent biological replicates were performed and the average and standard deviation were determined relative to the WT. Different letters above the bars represent statistically significant differences (*p*-value < 0.05). (**C**,**D**) Deletion of the high-affinity iron uptake system did not rescue *CCC1* overexpression toxicity. BY4741 (WT), *ccc1Δ*, *fet3Δ*, *ftr1Δ*, *aft1Δ*, and *cth2Δ* cells transformed with p416TEF (vector) or p416TEF-CCC1 (*P_TEF2_-CCC1*) were spotted on SC-Ura. Plates were incubated at 30 °C and photographed.

Previous studies demonstrated that overexpression of the vacuolar iron transporter *CCC1* increases iron uptake and total iron content, leading to its accumulation in the vacuole [8,9,25]. Given that iron is naturally detoxified and stored in the vacuole, we considered that overexpression of *CCC1* could be an appropriate strategy to increase yeast endogenous iron content. Therefore, we expressed the *CCC1* coding sequence under the control of the constitutive and high-expression promoter of *TEF2* gene (*P_TEF2_-CCC1*) in a *ccc1Δ* strain. Since *CCC1* deletion causes high cellular sensitivity to extracellular iron, we expected that *CCC1* overexpression would provide iron resistance [8,10]. To test yeast growth, we performed spot growth assays in solid media with increasing concentrations of ferrous ammonium sulfate (FAS). We used wild-type (WT) cells and *ccc1Δ* mutants as controls. As previously described, *ccc1Δ* cells (*ccc1Δ* + vector) grow similarly to WT cells (WT + vector) under iron-sufficient conditions (SC-Ura); however, they are more sensitive to an increase in extracellular iron, which limits *ccc1Δ* growth at 5 mM FAS and fully abrogates growth at 20 mM FAS (Figure 2A) [8]. Surprisingly, we observed that overexpression of *CCC1* with *TEF2* promoter was extremely toxic under normal iron-sufficient conditions, whereas growth was recovered upon addition of iron to the growth medium, resulting in higher resistance than *ccc1Δ* cells and only slightly less tolerance than WT cells (Figure 2A). These results indicate that altering the expression of *CCC1* under iron replete conditions could be detrimental to cells.

A potential explanation for the increase in iron acquisition and accumulation in the vacuole previously described for *CCC1*-overexpressing cells is an activation of the iron regulon due to decreased cytosolic and mitochondrial iron levels. Indeed, we observed that *P_TEF2_-CCC1* cells increased the expression of the members of the iron regulon, including the Aft1 targets *FTR1* and *CTH2*, as compared with WT cells (Figure 2B). Given that deletion of *FET3*, which encodes for an essential component of the cell surface Fet3-Ftr1 high-affinity iron transport complex, was shown to fully remove iron uptake of *CCC1*-overexpressing cells, we ascertained whether deletion of either *FET3* or *FTR1* would rescue its toxicity under iron-sufficient conditions [25]. As seen in Figure 2C, none of the deletions improved growth of *P_TEF2_-CCC1* cells, suggesting that other reasons beyond increased iron uptake and accumulation are responsible for *CCC1* toxicity under normal conditions (see Discussion). Consistent with this finding, decreasing extracellular bioavailability with the addition of an Fe^2+^-specific chelator (ferrozine, Fz) did not rescue growth of *P_TEF2_-CCC1* cells (Figure 2A, Fz). Previous data indicated that increased expression of *CTH2* under normal growth conditions also leads to cellular growth defects [33,34]. Since we observed that *CCC1* overexpression increases *CTH2* mRNA levels 7-fold (Figure 2B), we assessed whether deletion of *CTH2* rescued *CCC1* overexpression toxicity. As shown in Figure 2D, deletion of *CTH2* did not promote growth when *CCC1* is overexpressed, which indicates that the increased levels of *CTH2* are not the reason for toxicity. Next, we explored whether deletion of *AFT1* transcription factor, which would simultaneously limit iron uptake, iron mobilization from the vacuole, and *CTH2* expression, rescued the *CCC1* overexpression phenotype. Remarkably, we observed that instead of recovering growth, deletion of *AFT1* dramatically increased the toxicity of *CCC1* overexpression (Figure 2D). This result and the lack of rescue by *FTR1*, *FET3*, and *CTH2* deletions contradict the hypothesis that the underlying reason for *CCC1* toxicity is an increase in iron uptake, and strongly suggest that a decrease in cytosolic iron bioavailability, due to higher transport of iron into the vacuole mediated by Ccc1, is the most likely reason for this phenotype.

**Figure 3:**
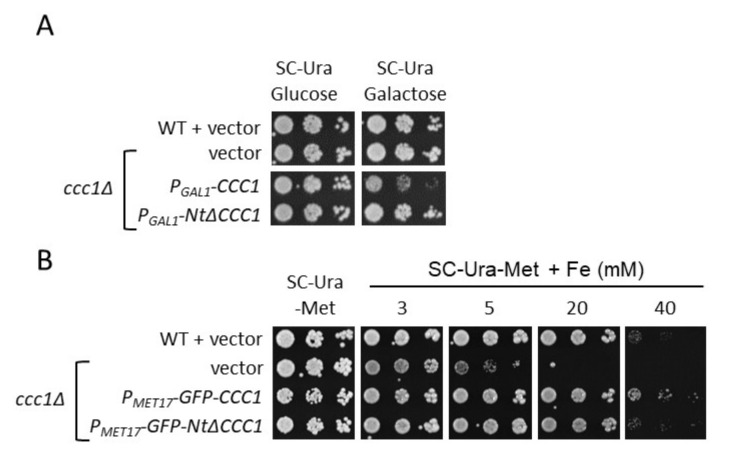
Growth of yeast cells expressing *CCC1* with regulatable promoters. (**A**) Expression of *CCC1*, but not *NtΔCCC1*, with *GAL1* promoter is toxic. BY4741 (WT) cells transformed with p416TEF (vector), and *ccc1Δ* cells transformed with p416TEF (vector), p416GAL-CCC1 (*P_GAL1_-CCC1*), and p416GAL-NtΔCCC1 were cultivated overnight in SC-Ura and then spotted on normal SC-Ura (SC-Ura Glucose) and SC-Ura without glucose but with 2% galactose (SC-Ura Galactose). (**B**) Expression of *CCC1* with *MET17* promoter rescues toxicity. BY4743 (WT) cells were transformed with p416TEF (vector), and *ccc1Δ* cells were transformed with p416TEF (vector), pUG36-CCC1 (*P_MET17_-GFP-CCC1*), and pUG36-NtΔCCC1 (*P_MET17_-*GFP-*NtΔCCC1*). Cells were cultivated overnight in SC-Ura (which contains methionine) to inhibit *CCC1* expression, and then spotted on SC-Ura-Met media with different concentrations of FAS (Fe). Plates were incubated at 30 °C and photographed.

To solve the toxicity caused by *CCC1* overexpression, we decided to test the regulatable promoters of *GAL1*, which is repressed by glucose and activated in media with galactose as the single carbon source (*P_GAL1_-CCC1* cells), and *MET17*, which is repressed by methionine (*P_MET17_-GFP-CCC1* cells). *P_GAL1_-CCC1* cells exhibited a strong toxicity when spotted on a galactose medium (SC-Ura/Galactose) (Figure 3A). This phenotype was probably due to excessive *CCC1* expression, as no growth defect was observed in SC-Ura (Figure 3A). Furthermore, *P_MET17_-GFP-CCC1* cells were cultivated in methionine-containing conditions to inhibit *CCC1* expression and then spotted on SC-Ura plates lacking methionine (SC-Ura-Met), which allows *CCC1* expression. Under these conditions, we did not observe any growth defect as compared with WT cells (Figure 3B), probably due to lower expression levels of *MET17*-driven expression as compared with *GAL1* and *TEF2* promoters. We noticed that *P_MET17_-GFP-CCC1* cells were as resistant to high iron concentrations as WT cells, which supports the notion that the GFP-Ccc1 tagged protein is functional (Figure 3B). Interestingly, iron resistance of *P_MET17_-GFP- NtΔCCC1* cells was lower than that of *P_MET17_-GFP-CCC1* cells, suggesting that the Ccc1 Nt region is important for function (Figure 3B). Moreover, these results indicate that the promoter that drives *CCC1* expression is critical for appropriate growth.

### 3.2. Deletion of CCC1 Amino-Terminal Region Rescues Toxicity by Limiting Iron-Resistance Capacity

Yeast *CCC1* protein contains an Nt region that faces the cytosol (Figure 1), and whose function has not been described. According to genome-wide data, this Nt domain contains multiple serine residues that are phosphorylated and lysine residues that are ubiquitylated [13,14]. To test whether this region was important for Ccc1 function under high-iron conditions or contributed to its toxicity under iron-sufficient conditions, we constructed a Ccc1 protein lacking its 87 Nt amino acids (NtΔCcc1). Remarkably, expression of *NtΔCCC1* with either *TEF2*, *GAL1*, or *MET17* promoter did not alter cell growth in iron-sufficient conditions (Figure 2A and Figure 3). A potential explanation for this result could be that deletion of the Nt region fully removes Ccc1 function, but this was not the case as both *P_TEF2_-NtΔCCC1* and *P_MET17_-NtΔCCC1* constructs conferred an iron resistant phenotype only slightly lower than that of the cells expressing *CCC1* under the control of the same promoter (Figure 2A and Figure 3B). Therefore, in contrast to full-length Ccc1, overexpression of *NtΔCCC1* does not cause cellular toxicity.

### 3.3. Cultures of NtΔCCC1 Cells Are More Efficient in Extracting Iron from Media

**Figure 4:**
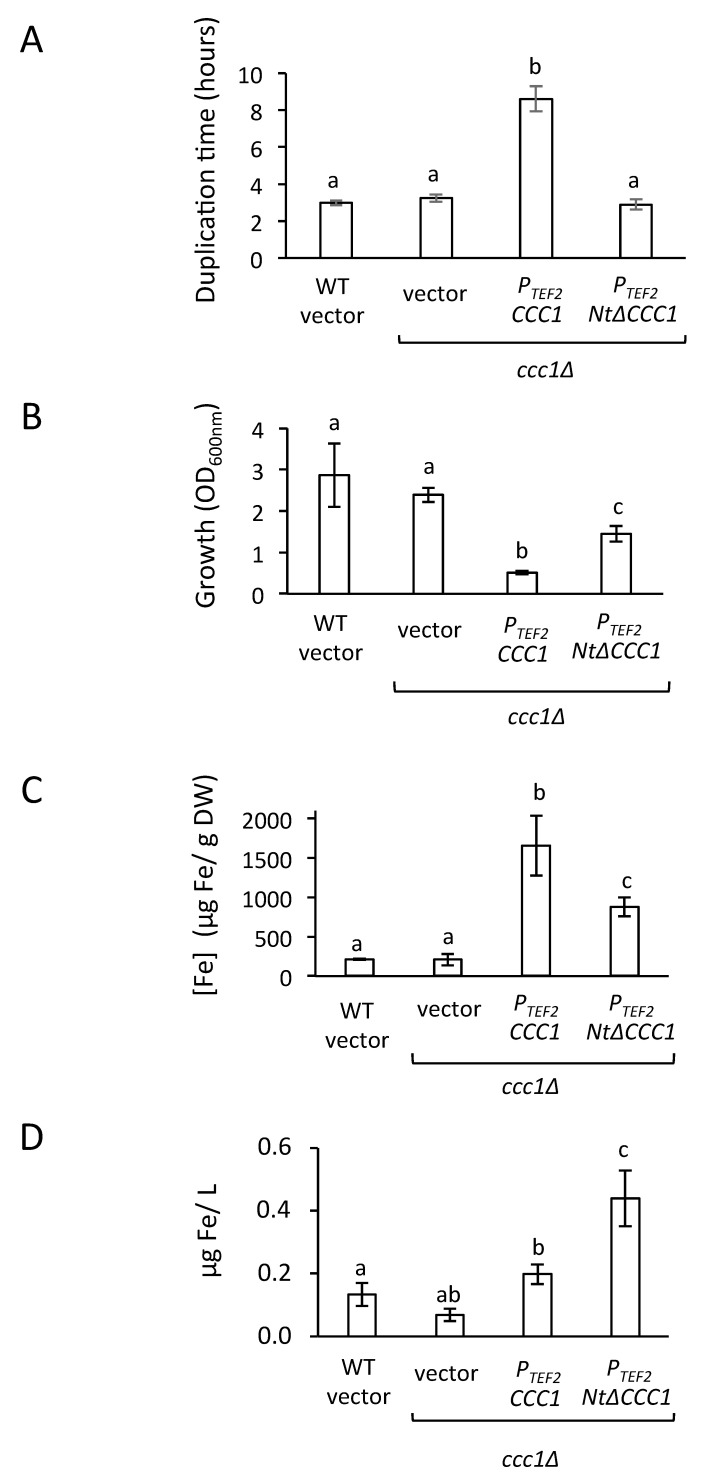
Overexpression of *NtΔCCC1* improves iron extraction from media. (**A**) Yeast cells described in Figure 2 were inoculated at an OD_600nm_ of 0.2 in SC-Ura with 500 μM FAS in a 96-well microplate reader and duplication times were calculated from the resulting growth curves as described in Materials and Methods. (**B**–**D**) Yeast cells described in Figure 2 were cultivated in SC-Ura + 500 μM FAS for 6 h, and OD_600nm_ (**B**), μg Fe/g dry weight (DW) (**C**), and μg Fe/L of medium (**D**) were determined. Three independent biological replicates were performed. The average and standard deviation were determined. Different letters above the bars represent statistically significant differences (*p*-value < 0.05).

To further study the contribution of the Ccc1 Nt region to iron acquisition and growth, we first cultivated WT, *ccc1Δ*, and cells expressing either *P_TEF2_-CCC1* or *P_TEF2_-NtΔCCC1* for 36 h in a microplate reader and calculated the duplication times from the resulting growth curves (Figure 4A and Figure S1). While we observed similar duplication times for WT, *ccc1Δ*, and *P_TEF2_-NtΔCCC1* cells, *P_TEF2_-CCC1* cells showed a 3-fold increase in duplication time, underscoring an important growth defect, in accordance with the results observed in solid media. Although *P_TEF2_-NtΔCCC1* cells showed a growth curve similar to those of WT and *ccc1Δ*, a small growth defect was indicated by the lower maximum OD_600nm_ achieved. To further characterize this differential growth, we then cultivated WT, *ccc1Δ*, and cells expressing either *P_TEF2_-CCC1* or *P_TEF2_-NtΔCCC1* for six hours in a liquid medium containing 500 μM FAS. We observed that WT and *ccc1Δ* cells grew properly, whereas *P_TEF2_-CCC1* cells again displayed a dramatic growth defect (Figure 4B). Remarkably, although *P_TEF2_-NtΔCCC1* cells grew better than *P_TEF2_-CCC1* cells did, they also exhibited a significant growth decrease as compared with WT and *ccc1Δ* cells (Figure 4B). Next, we measured cellular iron accumulation and observed an 8-fold and 4-fold increase in iron accumulation in *P_TEF2_-CCC1* and *P_TEF2_-NtΔCCC1* cells, respectively, as compared with WT cells (Figure 4C). These results suggest that *CCC1*-overexpressing cells could be used to extract and accumulate iron from the environment. However, when we determined total iron extracted per volume of medium, we observed that *P_TEF2_-CCC1* cells did not improve the capacity of WT or *ccc1Δ* cells to remove iron from the environmental milieu, mostly due to their low growth (Figure 4D). Interestingly, *P_TEF2_-NtΔCCC1* cells extracted twice as much iron from the extracellular medium as the rest of the cells assayed did (Figure 4D). These results suggest that overexpressing an Nt-truncated Ccc1 protein could be an interesting strategy to more efficiently obtain iron from media.

### 3.4. Ccc1 Amino-Terminus Modulates Subcellular Localization

**Figure 5:**
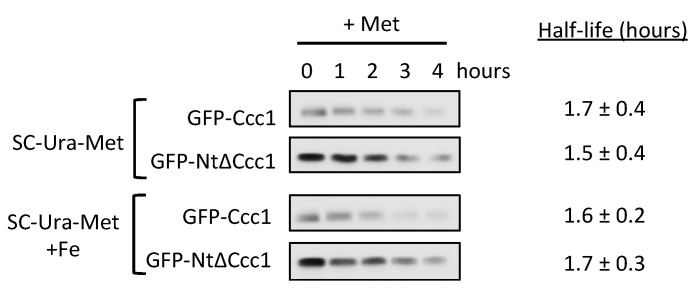
GFP-Ccc1 and GFP-NtΔCcc1 protein stability in normal and high-iron conditions. Yeast *ccc1Δ* cells transformed with either pUG36-CCC1 (*P_MET17_-GFP-CCC1*) or pUG36-NtΔCCC1 (*P_MET17_-*GFP-*NtΔCCC1*) plasmids were cultivated in SC-Ura-Met to exponential phase and 3 mM FAS was added (or not) for 1 h. Expression was stopped by addition of 3 mM methionine, and aliquots were isolated at the indicated time points. Total proteins were extracted, and Ccc1 protein levels were determined by immunoblotting with anti-GFP antibody. Equal amounts of total proteins were loaded in each lane. Three independent biological experiments were performed, and GFP-Ccc1 and GFP-NtΔCcc1 protein half-lives was determined. The average half-life and its standard deviation are presented.

The decreased resistance to high-iron conditions of yeast cells expressing *NtΔCCC1* could be due to decreased stability or mislocalization of the truncated protein. Moreover, Ccc1 Nt contains serine and lysine residues that, according to genome-wide studies, are phosphorylated and ubiquitylated, respectively (Figure 1) [13,14]. To ascertain if this was the case, we used yeast cells expressing GFP-tagged Ccc1 or NtΔCcc1 proteins under the control of *MET17* promoter, previously assayed for growth in iron-containing media (Figure 3B). It is important to highlight that incorporation of an Nt tag, such as GFP, precludes any change in protein stability due to differences in the initial amino acids of the protein, allowing direct conclusions to be made from the Nt region. Thus, we investigated protein stability by stopping *CCC1* and *NtΔCCC1* expression with the addition of methionine and determining protein levels at different time points. As shown, Ccc1 protein stability was not altered by the deletion of its Nt domain, neither under normal growth conditions nor after addition of iron to the medium (Figure 5 and Figure S2). These results indicate that the deleted Nt domain does not significantly alter Ccc1 protein stability under these growth conditions.

**Figure 6:**
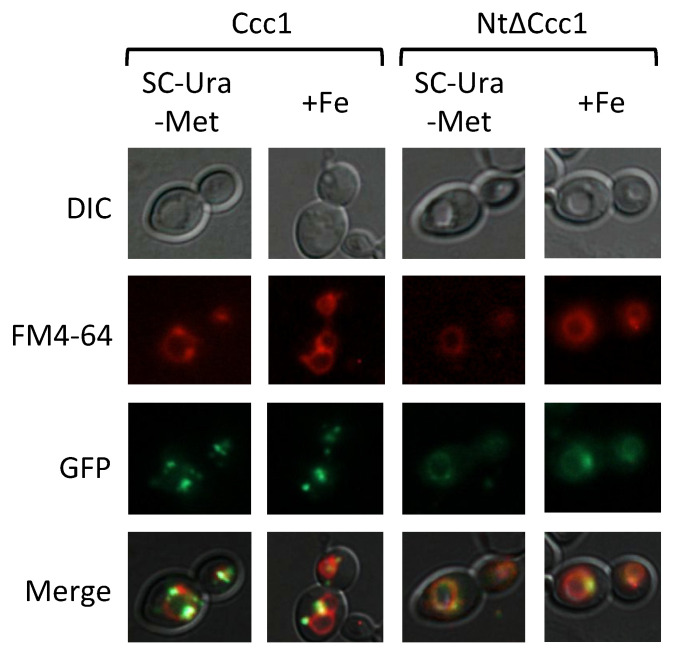
Subcellular localization of Ccc1 and NtΔCcc1 proteins in normal (SC-Ura-Met) and high-iron (+Fe) conditions. Yeast cells were cultivated as described in Figure 5, and visualized under Nomarski (DIC) and GFP fluorescence optics. Vacuolar membranes were stained with the styryl dye FM4-64 as indicated in Materials and Methods. Merge shows the overlap of the different signals.

To ascertain whether deletion of Ccc1 Nt region alters cellular distribution, we determined the subcellular localization of GFP-Ccc1 and GFP-NtΔCcc1 proteins by fluorescence microscopy under iron-sufficient and high-iron conditions (1 h in 3 mM FAS). Differential interference contrast (DIC) was used to visualize vacuoles, since they appear as clear indentations, whereas the lipophilic dye FM4-64 selectively localized to vacuolar membranes after appropriate incubation. We observed that full length Ccc1 protein colocalized with FM4-64 under both iron-sufficient and high-iron conditions, particularly at specific regions of the vacuolar membrane (Figure 6). NtΔCcc1 protein displayed a more uniform distribution throughout the vacuolar membrane, as compared with wild-type Ccc1 under both iron-sufficient and high-iron conditions (Figure 6). These results suggest that the Ccc1 Nt domain is important in modulation of Ccc1 protein localization.

## 4. Discussion

An important drawback of increasing intracellular iron concentrations is the toxicity caused by ROS generation. Here, we decided to explore the potential of the main yeast iron resistance factor, Ccc1, to increase endogenous iron by storing it in an unharmful form within the vacuole. For this purpose, we overexpressed *CCC1* and observed that it led to low growth and cellular viability when cells were cultivated in normal iron replete media (Figure 2 and Figure 3). Since increased expression of *CCC1* stimulates iron uptake and accumulation due to iron regulon activation (Figure 2), we postulated that the reason for Ccc1 toxicity could be iron acquisition [8,9,25]. In fact, previous genetic and biochemical analyses demonstrated that iron toxicity was due to the accumulation of cytosolic iron [35]. Moreover, the toxicity caused by the expression of a constitutively active Aft1 transcription factor was shown to be, at least partially, rescued by deletion of *FET3* [36]. However, deletion of the components of the high-affinity iron uptake system Fet3-Ftr1 at the cell surface did not rescue the growth of *CCC1*-overexpressing cells (Figure 2). We investigated whether the upregulation of *CTH2*, which was previously shown to limit growth, was responsible for *CCC1* overexpression toxicity, but no rescue was observed in a *cth2Δ* mutant (Figure 2) [33,34]. Unexpectedly, we observed that deletion of the *AFT1* transcriptional factor increased *CCC1* overexpression toxicity (Figure 2). Aft1 is mainly implicated in increasing cytosolic iron levels by promoting iron uptake and mobilization from the vacuole. Therefore, these results are consistent with *CCC1* causing a decrease in the bioavailability of iron to the cytosol and other organelles, including mitochondria, whose pools are highly interconnected [37,38]. Indeed, the activation of *FTR1* and *CTH2* that we observed (Figure 2A) is probably a consequence of decreased iron-sensing by Aft1 transcription factor. In any case, we cannot discard other non-exclusive possibilities for such toxicity. For instance, *CCC1* overexpression could cause a decrease in vacuolar lumen acidification, since Ccc1 seems to be a H^+^/Fe^2+^ antiporter [12]. In this sense, recent data demonstrated that the loss of vacuolar acidification leads to oxidative damage of mitochondrial Fe/S cluster biosynthesis components and activities and the development of an age-related mitochondrial dysfunction [39,40]. Importantly, Ccc1 overexpression toxicity was not observed in high iron media (Figure 2 and Figure 3). This result is consistent with the natural expression pattern of *CCC1*, which is induced in response to elevated extracellular iron, and reinforces the hypothesis that low cytosolic and mitochondrial iron levels could be the reason for its toxicity [18,20]. However, more detailed studies are necessary to fully elucidate the molecular reason for the toxicity of Ccc1 protein when overexpressed under iron replete conditions.

The comparison of Ccc1/VIT1 protein sequences unveiled that their cytosolic Nt domain was not conserved during evolution and displays particular characteristics depending on the species [11]. In the case of fungi, including *S. cerevisiae*, Ccc1 Nt is rich in proline and serine residues [11]. The fact that genome-wide studies show that these serine residues could be phosphorylated prompted us to explore Ccc1 Nt function by constructing and expressing an NtΔCcc1 protein lacking its first 87 amino acids [13,14]. Overexpression of *NtΔCCC1* did not confer toxicity under any of the promoters assayed (Figure 2 and Figure 3). This was probably due a decrease in NtΔCcc1 functionality since it did not produce the same resistance to high iron media as full length Ccc1 did (Figure 2 and Figure 3). We observed that both GFP-tagged Ccc1 and NtΔCcc1 proteins localized to the vacuolar membrane, although full-length Ccc1 preferentially accumulated in small regions of the vacuolar membrane (Figure 6). It is tempting to speculate that phosphorylation of serine residues within the Ccc1 Nt domain could modulate subcellular distribution of the protein. It was proposed that Ccc1/VIT1 proteins dimerize, but no aggregation of such proteins has been described [12]. Further studies are necessary to decipher the contribution of the Nt domain to Ccc1 function in metal transport. Importantly, NtΔCcc1-expressing cells were still able to upregulate iron incorporation while maintaining considerable growth (Figure 4). Consequently, NtΔCcc1 cells were able to incorporate at least 2-fold higher levels of iron from iron replete media than WT, *ccc1Δ*, and *CCC1*-overexpressing cells were. 

## 5. Conclusions

Iron deficiency is a prevalent nutritional disorder whose prevention requires the development of novel strategies that increase iron intake. Baker’s yeast *S. cerevisiae* is widely used in food technology to produce bakery derived products but also as a food supplement. Therefore, greater knowledge of yeast iron homeostasis and its ability to increase its capacity to acquire and store iron could prove meaningful. Here, we have observed that overexpression of the vacuolar iron importer Ccc1 is toxic to yeast cells. Remarkably, overexpression of an Nt-truncated version of Ccc1 (NtΔCcc1) did not alter growth and led to an increase in the extraction of iron from the environmental medium. Therefore, this truncated version of Ccc1 protein could be used to design strategies aimed to increase the capacity of yeast cells to incorporate iron from non-iron rich media. Additional studies aiming to enrich yeast cells with iron could contribute to its utilization as a tool to palliate iron deficiency.

## Data Availability

The data presented in this study are openly available in Digital.CSIC (https://digital.csic.es, accessed on 13 July 2021) at DOI: http://dx.doi.org/10.20350/digitalCSIC/13962 (accessed on 13 July 2021).

## Supplementary Materials

The following are available online at https://www.mdpi.com/article/10.3390/genes12081120/s1, Figure S1: Overexpression of CCC1 induced a decrease in the growth rate. Yeast cells described in Figure 2 were inoculated at an OD600nm of 0.2 in liquid SC-Ura medium supplemented with 500 µM FAS. The OD600nm was recorded every 30 minutes with a Spectrostar Nano absorbance 96-plate reader for 36 h at 28 °C. The average curve and standard deviation of at least three independent biological replicates are shown. Figure S2. GFP-Ccc1 and GFP-Nt Ccc1 protein stability quantification and loading controls. (A) Quantification of GFP-Ccc1 and GFP-Nt Ccc1 protein levels. The average of three different samples and their standard deviation is presented. (B) Western blot for Pgk1 protein levels. (C) Ponceau staining.
