# Supplementary material for: Expression of a Truncated Yeast Ccc1 Vacuolar Transporter Increases the Accumulation of Endogenous Iron
Source: Genes (Basel). 2021 Jul 23;12(8):1120. doi: 10.3390/genes12081120 (PMC8391176; doi:10.3390/genes12081120)
Supplement: Supplementary file 1 [file genes-12-01120-s001.zip › genes-1271672-supplementary.pdf]

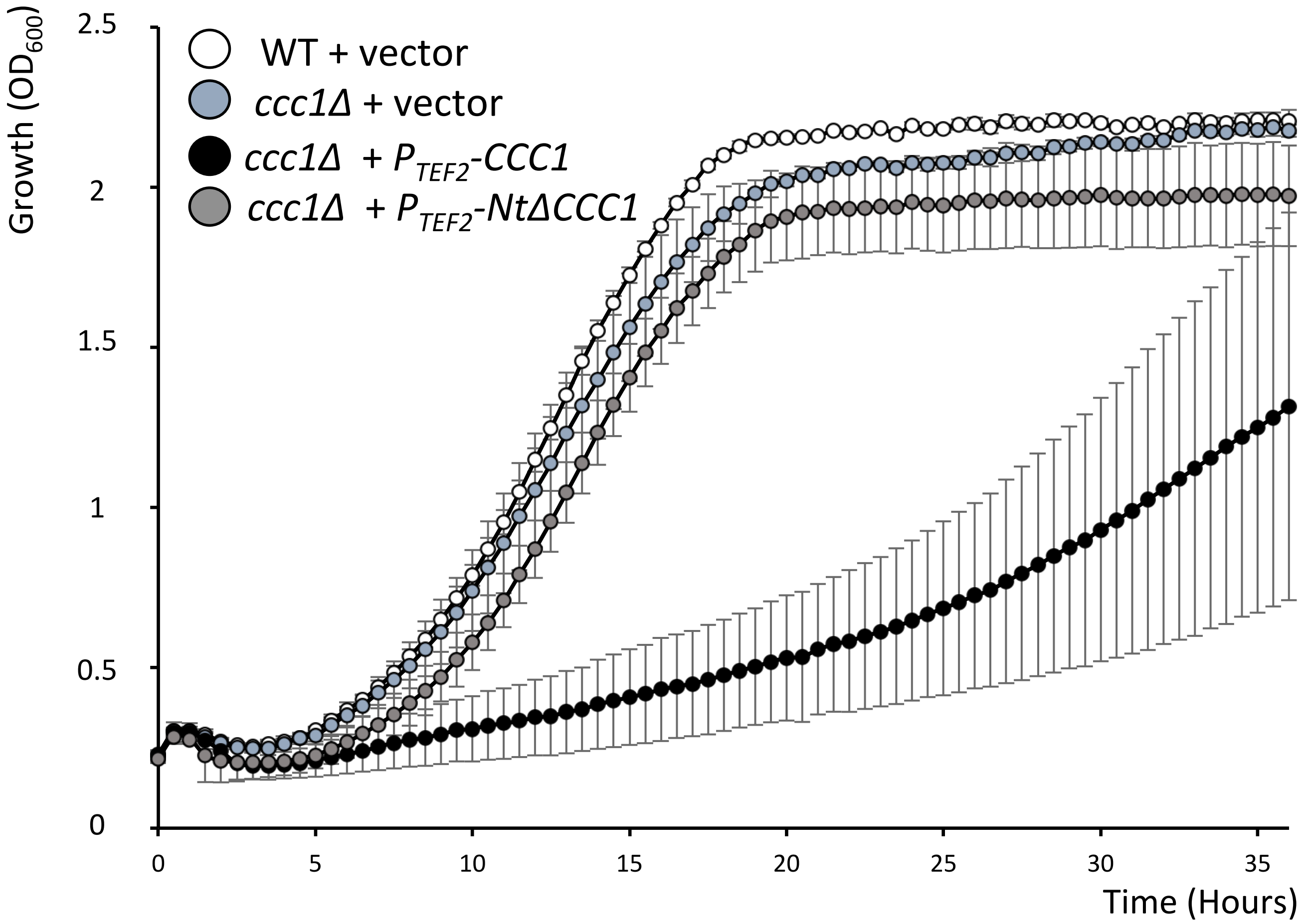


**Figure S1.** Overexpression of *CCC1* induces a decrease in the growth rate. Yeast cells describred in Figure 2 were inoculated at an $OD_{600}$ of 0.2 in liquid SC-ura medium supplemented with 500 µM FAS. The $OD_{600}$ was recorded every 30 minutes with a Spectrostar Nano absorbance 96-plate reader for 36 hours at 28°C. The average curve and standard deviation of at least three independent biological replicates is shown.

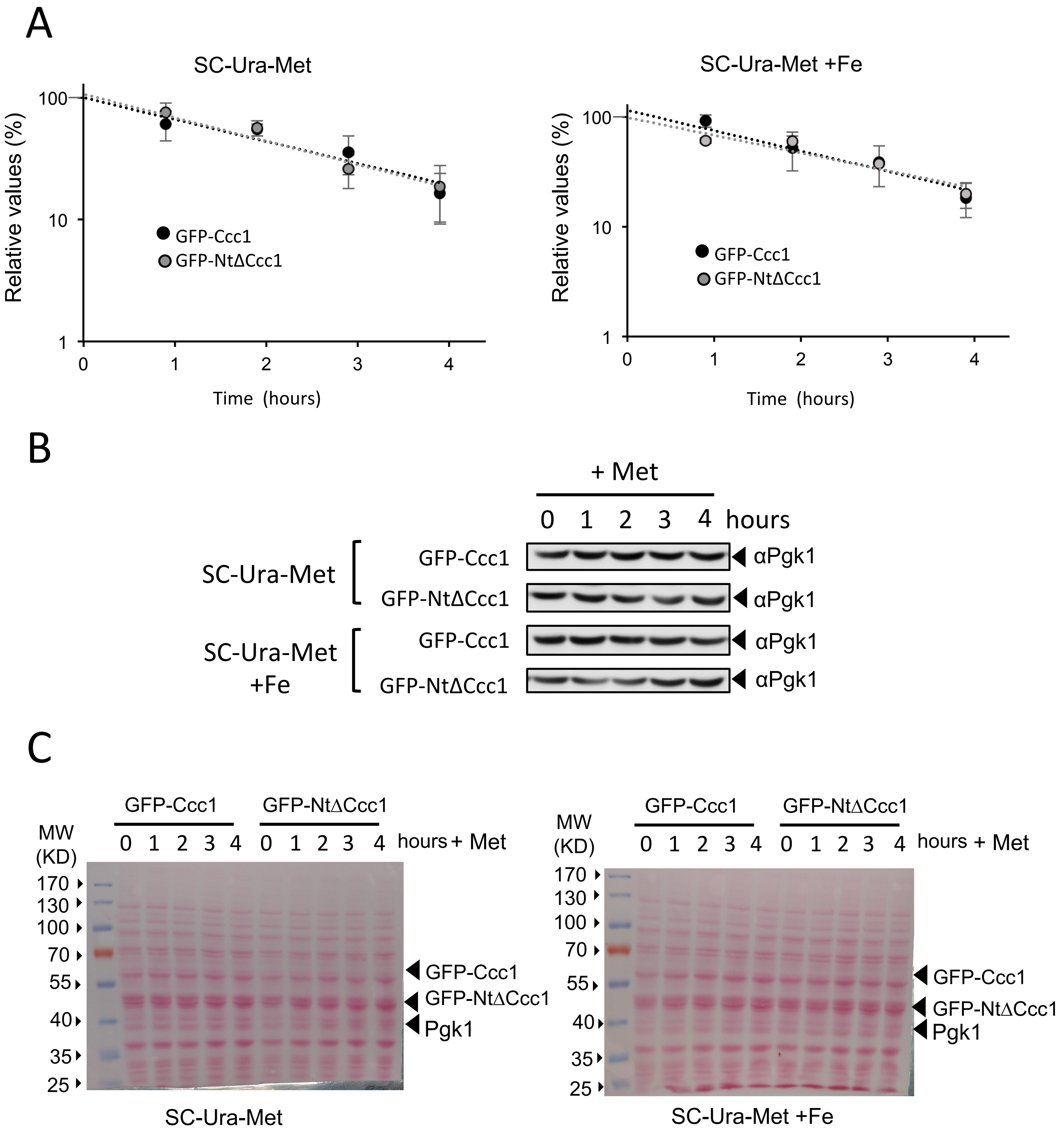


**Figure S2.** GFP-Ccc1 and GFP-NtΔCcc1 protein stability quantification and loading controls. (**a**) Quantification of GFP-Ccc1 and GFP-NtΔCcc1 protein levels. The average of three different samples and its standard deviation is represented. (**b**) Western blot for Pgk1 protein levels. (**c**) Ponceau staining.
